# Video Games and Gamification for Assessing Mild Cognitive Impairment: Scoping Review

**DOI:** 10.2196/71304

**Published:** 2025-08-05

**Authors:** Yu Chen, Kathrin Gerling, Katrien Verbert, Vero Vanden Abeele

**Affiliations:** 1 e-Media Research Lab Faculty of Engineering Technology KU Leuven Leuven Belgium; 2 Augment Group Department of Computer Sciences KU Leuven Leuven Belgium; 3 Karlsruhe Institute of Technology Karlsruhe Germany

**Keywords:** mild cognitive impairment, video games, gamification, digital biomarkers, cognitive function assessment, artificial intelligence, AI

## Abstract

**Background:**

Early assessment of mild cognitive impairment (MCI) in older adults is crucial, as it enables timely interventions and decision-making. In recent years, researchers have been exploring the potential of gamified interactive systems (GISs) to assess pathological cognitive decline. However, effective methods for integrating these systems and designing GISs that are both engaging and accurate in assessing cognitive decline are still under investigation.

**Objective:**

We aimed to comprehensively investigate GISs used to assess MCI. Specifically, we reviewed the existing systems to understand the different game types (including genres and interaction paradigms) used for assessment. In addition, we examined the cognitive functions targeted. Finally, we investigated the evidence for the performance of assessing MCI through GISs by looking at the quality of validation for these systems in assessing MCI and the diagnostic performance reported.

**Methods:**

We conducted a scoping search in IEEE Xplore, ACM Digital Library, and Scopus databases to identify interactive gamified systems developed for assessing MCI. Game types were categorized according to genres and interaction paradigms. The cognitive functions targeted by the systems were compared with those assessed in the Montreal Cognitive Assessment (MoCA). Finally, we examined the quality of validation against the reference standard (ground truth), relevance of controls, and sample size. Where provided, the diagnostic performance on sensitivity, specificity, and area under the curve was reported.

**Results:**

A total of 81 articles covering 49 GISs were included in this review. The primary game types used for MCI assessment were classified as casual games (30/49, 61%), simulation games (17/49, 35%), full-body movement games (4/49, 8%), and dedicated interactive games (3/49, 6%). Of the 49 systems, 6 (12%) assessed cognitive functions comprehensively, compared to those functions assessed via the MoCA. Of the 49 systems, 14 (29%) had validation studies, with sensitivities ranging from 70.7% to 100% and specificities ranging from 56.5% to 100%. The reported diagnostic performances of GISs were comparable to those of common screening instruments, such as Mini-Mental State Examination and MoCA, with some systems reporting near-perfect performance (area under the curve>0.98). However, these findings often stemmed from small samples and retrospective designs. Moreover, some of these systems’ model training and validation exhibited substantial deficiencies.

**Conclusions:**

This review provides a comprehensive summary of GISs for assessing MCI, exploring the cognitive functions assessed by these systems and evaluating their diagnostic performance. The results indicate that current GISs hold promise for the assessment of MCI, with several systems demonstrating diagnostic performance comparable to established screening tools. Nevertheless, despite some systems reporting impressive performance, there is a need for improvement in validation, particularly concerning sample size and methodological rigor. Future work should prioritize prospective validation and present greater methodological consistency.

## Introduction

### Background

Cognitive impairment refers to the decline of one or more 1 cognitive functions, including attention, executive function, or memory [1]. This decline may be a natural part of aging [2]. However, with the increase in average lifespan, the prevalence of *pathological* cognitive decline also rises due to excessive neural damage [3]. This pathological decline is known as dementia, a general term for a variety of neurodegenerative diseases with different causes characterized by “an impaired ability to remember, think, or make decisions interfering with daily activities” [4]. Most types of dementia develop gradually over time [5]. The prestage of dementia is called mild cognitive impairment (MCI). More particularly, MCI is defined as “a syndrome of self-reported cognitive complaint with one or more objective cognitive impairments but preserved independence in functional abilities” [6]. Hence, individuals with MCI, despite experiencing cognitive impairment, still successfully perform activities of daily living (cooking, toileting, walking, visiting friends, etc), and not all individuals with MCI go on to develop dementia [6]. However, they are at a higher risk, with a reported annual conversion rate from MCI to dementia varying from 2% to 31% [7].

Currently, pharmaceutical interventions to cure MCI or dementia are still under research [8]. Nevertheless, early assessment of cognitive decline, along with early intervention methods, such as cognitive training and medication, can effectively slow down the progression of the condition [9-12]. The current gold standard for diagnosing MCI involves a detailed anamnesis, followed by a neuropsychological examination, and is often complemented by brain imaging to detect structural changes, along with blood and cerebrospinal fluid analyses [13,14]. This comprehensive assessment necessitates a team of medical specialists, consuming a significant number of medical resources [15]. Therefore, before a detailed examination is performed, it is advised to administer a quicker screening test and to continue with a full examination only if necessary [16]. The Mini-Mental State Examination (MMSE) [17] is currently the most widely used instrument for rapid screening of Alzheimer disease (AD) and MCI [18], assessing cognitive functions such as attention, registration and recall, orientation, and language. It is fast to administer, taking only 10 minutes, but it has also shown a lack of sensitivity, misclassifying older adults with MCI as healthy [19]. The Montreal Cognitive Assessment (MoCA) [20] is another popular instrument that evaluates similar cognitive functions in up to 20 minutes. Several studies have shown MoCA to be superior to the MMSE in terms of sensitivity for detecting MCI [20-22]. However, the MoCA has shown lower specificity, misclassifying healthy older adults (with natural cognitive decline) as at risk for MCI.

The suboptimal accuracy of these quick screening instruments may be partly due to the limited assessment time inherent in their design. However, another factor negatively impacting the accuracy of neuropsychological tests may be anxiety. Patients who are required to perform abstract tasks at a clinic in the presence of medical experts may experience heightened stress levels and perform suboptimally [23]. This is known as the white coat effect [24] and is known to disturb neuropsychological test performance. Moreover, neuropsychological tests are designed as one-time assessments and can be disturbed by circumstances such as lack of sleep or traumatic events (eg, loss of a spouse). However, the already long waiting lists make frequent and repeated neuropsychological tests infeasible.

Therefore, to complement screening instruments for MCI and alleviate the resources needed for a full examination, researchers are exploring alternative sources of information on cognitive functioning. Such solutions may come in the form of dedicated sensors installed in the home or wearables to detect movements [25,26], embedded software trackers that capture mouse movements [27,28], keyboard loggers installed on computers [29], conversational humanoids [30], or the use of interactive virtual simulations and games. Particularly, the latter has stirred the interest of researchers; games and simulations have long been used in understanding, measuring, and training cognition [31-33]. In addition to providing interactive, immersive environments to engage participants, they provide challenges bounded by a fixed set of rules, which can be highly indicative of targeted cognitive functioning. Research has indicated that games are more engaging than classical neuropsychological tests [32-34] and can be used to assess cognitive skills [35]. Moreover, the entertaining nature of gamified interactive systems (GISs) might reduce stress. They can be played in the comfort of the patient’s home, making the presence of a trained administrator redundant [36]. Finally, GISs also encourage more frequent interaction, providing additional testing data. Thus, they have the potential to *complement* traditional screening instruments by offering longitudinal assessment, which provides health care professionals with a new perspective for their case findings and diagnosis.

Despite their potential, it remains unclear to what extent GISs can be effectively used for the assessment of MCI, particularly as an early indicator of dementia. Researchers are still investigating the effective methods for system integration [37] and the accuracy of GISs in assessing cognitive decline [31,37]. In summary, while the of GISs for assessing MCI holds potential clinical significance, it remains in its early stages of development.

### Objectives

Therefore, to provide a more comprehensive understanding of the potential of GISs, we aimed to present a scoping review of their applications in assessing MCI, addressing the following research questions (RQs).

1. What are the different game types (genres and interaction paradigms) of GISs used to assess MCI?
2. Which cognitive functions are assessed by GISs?
3. How are GISs evaluated (eg, longitudinal or cross-sectional), and how reliable are these studies in reporting diagnostic performance?

## Methods

### Overview

The review followed the latest PRISMA-ScR (Preferred Reporting Items for Systematic Reviews and Meta-Analyses extension for Scoping Reviews) guidelines (checklist 1) [38], focusing on GISs for assessing MCI. The protocol is provided in Multimedia Appendix 1 [9-12,20-22,38,39] and can also be accessed online [40]. The databases queried include the Scopus database (eg, PubMed, MEDLINE, and Embase) as well as more technical libraries, specifically IEEE Xplore and ACM Digital Library. The screening process for queried articles consisted of 3 phases: title screening, abstract screening, and full-text eligibility. Ambiguous articles at each phase were advanced to the next stage for further review. In the full-text eligibility, articles were reassessed to ensure that all 3 inclusion criteria were met, in addition to the 4 exclusion criteria.

### Search Queries Used

The query terms used in the search strategy are presented in Textbox 1. For the population, the search focused on patients with either “Mild Cognitive Impairment” or “MCI,” deliberately excluding dementia to maintain an emphasis on the prestage of dementia. Regarding the instrument used for assessment, the search included the terms “Gamif*,” “Game,” “Video game,” or “Videogame.” In addition, the purpose of the instrument was specified as “assessment,” with variations such as “Evaluation,” “Screen*,” and “Diagnos*” included as synonyms in the queries. These terms were searched across metadata, titles, abstracts, keywords, and full text. Because the syntax of each database varied slightly, the queries were adapted to match the specific requirements of each library, with the exact queries provided in the Multimedia Appendix 2.

**Summary of search terms.**

**Disease**

- Mild Cognitive Impairment
- MCI

**Instrument**

- Gamif*
- Game
- Video game
- Videogame

**Purpose**

- Assess*
- Evaluat*
- Measure*
- Screen*
- Diagnos*

### Eligibility Criteria

We focused on GISs designed to assess MCI and their potential for such assessment in this review. As a result, only articles meeting the predefined inclusion and exclusion criteria were included, as detailed in Textbox 2.

For systems described in multiple articles, such as separate publications for system design and diagnostic performance, all related articles were included and considered collectively as the entirety of a system. For systems that the authors named, we use the name in the remainder of the paper. For those systems that were not named by the authors, we refer to the system number given in this review.

**Inclusion and exclusion criteria.**

**Inclusion criteria**

- Full-text, peer-reviewed articles published in English
- Systems assessing mild cognitive impairment (MCI) as part or all of their function
- Explicit inclusion of game or gamified elements in the article

**Exclusion criteria**

- Studies featuring games or gamified elements in nonelectronic forms, such as pen-and-paper games
- Game or gamified systems designed only for rehabilitation or training of specific cognitive functions, or targeting dementia instead of MCI
- Articles that did not claim implementation
- Book chapters that are inaccessible or retracted articles

### Data Extraction and Synthesis

The following subsections describe how data were extracted and analyzed for each RQ, in alignment with the Results section by RQs.

#### RQ1: Identifying Game Types and Interaction Paradigms

We first extracted the information needed to address RQ1*.* We described the GISs used in articles based on genre (eg, casual games or simulation games [41]) and interaction paradigm [42].

#### RQ2: Mapping Cognitive Functions to the MoCA Domains

Second, we extracted the information needed to address RQ2*.* We drew on the descriptions provided in each paper and mapped the mentioned cognitive functions to those assessed by the MoCA [20], as outlined in Textbox 3.

We chose MoCA over the MMSE because it provides a more sensitive and comprehensive assessment of MCI [43], particularly in domains relevant to many GISs [44]. MoCA includes dedicated tasks for executive functions (eg, trail making, verbal fluency, and abstraction) and more detailed visuospatial assessments (eg, cube copying and clock drawing) than MMSE. These domains are frequently targeted by GISs, making MoCA a more suitable framework for mapping cognitive coverage.

**Mapping of cognitive functions to the Montreal Cognitive Assessment domains.**

**Test**

- Short-term memory: assesses the ability to recall information after a short delay.
- Visuospatial abilities: evaluates skills such as drawing and understanding spatial relationships.
- Executive functions: tests planning, problem-solving, and organizing abilities.
- Attention, concentration, and working memory: measures the ability to focus, maintain attention, and manipulate information.
- Language: assesses naming, fluency, and comprehension.
- Orientation to time and place: evaluates awareness of current date, location, and situation.

#### RQ3: Evaluation Design, Quality Assessment, and Diagnostic Performance Reporting

Finally, we extracted the information to address RQ3*.* Here, only systems that included evaluations for assessing MCI were coded. Each system may have multiple studies encompassing different research designs that have been published. If multiple studies of 1 system were included in the review, we chose articles focusing on the report of diagnosis. Usability studies, or studies focused on collecting other data (eg, physiological signals), were not considered to answer RQ3.

We first investigated *how GISs are evaluated.* Therefore, we coded the different research study designs used to assess accuracy, for example, cross-sectional studies or longitudinal studies. Second, we investigated *how reliable these studies are.* The articles were evaluated across 3 dimensions, each scored on a scale from 1 to 3, with higher scores indicating higher quality, based on a quality assessment method adapted from the studies by Bezabih et al [45], Johnson et al [46], and Boyle et al [47].

The detailed dimensions and scoring methods are provided in Textbox 4.

Hence, the score for each article can range from 4 to 12. We consider scores from 3 to 5 as “weak evidence,” 6 to 7 as “moderate evidence,” and scores 8 to 9 as “strong evidence.”

Finally, we investigated the *diagnostic performances that were reported.* The actual outcomes of the studies were coded, including sensitivity, specificity, and area under the curve (AUC).

**Dimensions and scoring methods.**

**How appropriate is the reference standard? (1 to 3 out of 3 points)**

1. Reference standard is based on fast screening instruments (eg, Mini-Mental State Examination or Montreal Cognitive Assessment)
2. Reference standard is based on a comprehensive neuropsychological test
3. Reference standard is based on full diagnosis by clinicians

**How relevant are the comparative groups? (1 to 3 out of 3 points)**

1. No controls, or it is unspecified who or how many participants have mild cognitive impairment (MCI)
2. MCI versus healthy controls, MCI versus older adults with subjective cognitive decline, or MCI versus older adults with dementia MCI versus healthy controls, with additional subdistinctions, for example, additional persons with subjective cognitive decline or persons with Alzheimer disease, or intraperson measurement (in case of long-term measurement in persons with MCI)

**How generalizable are the findings of this study to the target population, considering the method of analysis and sample size of persons with MCI? (1 to 3 out of 3 points; for studies other than cross-sectional studies, the score was given case by case)**

1. Cross-sectional study with <10 participants with MCI
2. Cross-sectional study with 10 to 30 participants with MCI
3. Cross-sectional study with >30 MCI participants with MCI

## Results

### Overview

**Figure 1:**
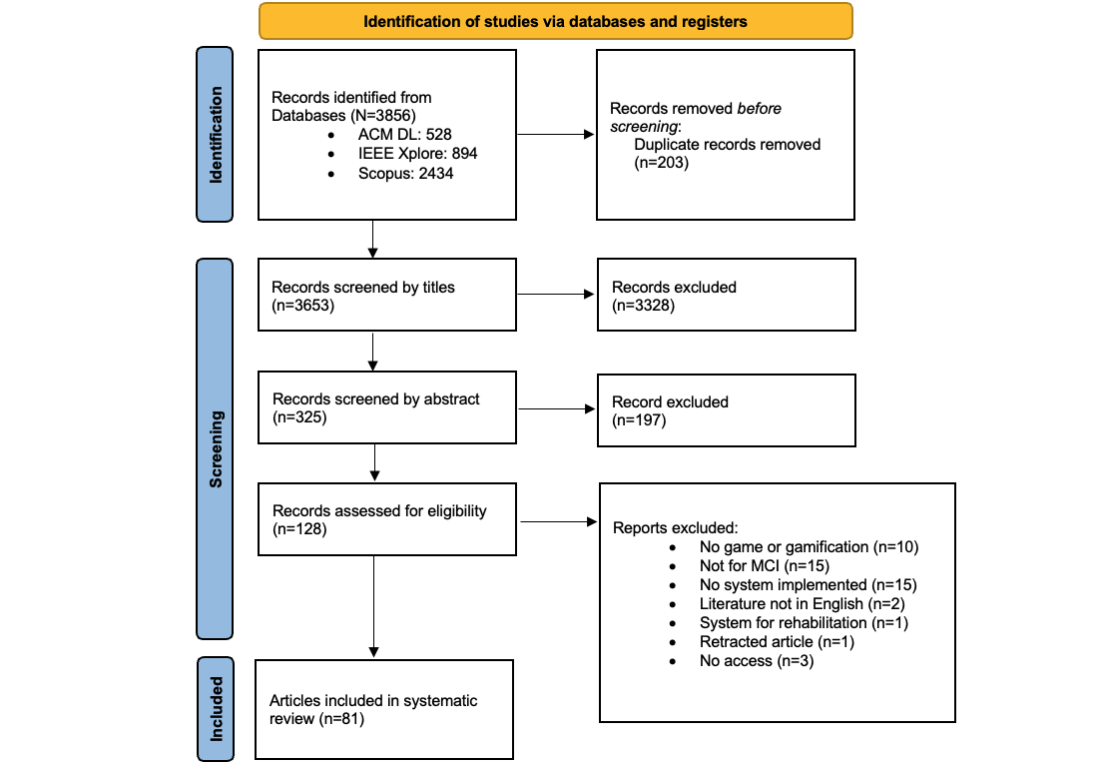
Screening and inclusion process. DL: Digital Library; MCI: mild cognitive impairment.

The search was executed in July 2024. A total of 3856 articles were retrieved based on the queries listed in Textbox 1. The specific numbers from each database are detailed in Figure 1. Before further screening, 5.26% (203/3856) of duplicate articles were removed. After the title and abstract screening, 96.5% (3525/3653) of the articles were excluded, leaving 3.5% (128/3653) of the articles for full-text evaluation. As a result, 47 (36.7%) of the 128 articles were excluded for reasons outlined in Figure 1. Ultimately, this scoping review included 81 (63.3%) of the 128 articles describing 49 GISs, as shown in Table 1 [48-128].

**Table 1:** Summary of included studies.

System number	Name of the gamified interactive system	Reference
Sys1	—a	[48-50]
Sys2	Fun Cube	[51,52]
Sys3	VAP-Sb	[53,54]
Sys4	VREAD	[55,56]
Sys5	CWGc and CSGd	[57,58]
Sys6	SmartAgeing	[59-64]
Sys7	FitForAll	[65-67]
Sys8	Kitchen and Cooking	[68]
Sys9	Find the Pair	[69]
Sys10	VAP-Me	[70]
Sys11	Whack-a-Mole	[71-73]
Sys12	—	[74]
Sys13	Smartkuber	[75]
Sys14	—	[76,77]
Sys15	—	[78]
Sys16	—	[79]
Sys17	Dr. Solitaire	[80-83]
Sys18	—	[84]
Sys19	Counting sheep	[85,86]
Sys20	Panoramix	[87-92]
Sys21	Neuro-World	[93,94]
Sys22	—	[95]
Sys23	Virtual ADL+House	[96]
Sys24	—	[97]
Sys25	RE@CH	[98]
Sys26	WarCAT	[99]
Sys27	—	[100]
Sys28	Holey Moley	[101]
Sys29	Hit-the-ball	[102]
Sys30	Virtual Supermarket	[103-106]
Sys31	—	[107]
Sys32	VSIDCSf	[108]
Sys33	Lucy	[109,110]
Sys34	Quick, Draw!	[111]
Sys35	—	[112]
Sys36	COGNIPLAT	[113,114]
Sys37	Pac-man	[115]
Sys38	CogWorldTravel	[116,117]
Sys39	—	[118]
Sys40	Neurocity	[119]
Sys41	Minecraft	[120]
Sys42	Seas the Day	[121]
Sys43	—	[122]
Sys44	—	[123]
Sys45	—	[124]
Sys46	BrightArm	[125]
Sys47	RehabCity	[126]
Sys48	The Ryokansan	[127]
Sys49	X-Torp	[128]

^a^VAP-S: virtual action planning supermarket.

^b^VAP-M: Virtual Action Planning Museum.

^c^Not available.

The results of the articles included are presented in the following sections, organized according to the RQs.

### RQ1: What Are the Different Game Types (Genres and Interaction Paradigms) of GISs Used to Assess MCI?

When reviewing GISs according to genre and interaction paradigm, we identified GISs to be casual games, simulation games, full-body movement games, or dedicated interactive games, as illustrated in Multimedia Appendix 3.

#### Casual Games

#### Overview

Casual games (30/49, 61%) are a category of video games known for their accessibility and ease of play. These games are designed to be simple to learn, offering quick rewards and a forgiving gameplay experience, ultimately creating a fun and enjoyable entertainment experience for players of all skill levels [129]. As for the interaction paradigm, casual games run on general digital devices, such as smartphones, tablets, and PCs, without a need for specific controllers. A keyboard or a computer mouse, or simply a touch screen, is sufficient. We further categorized casual games into the following subcategories.

#### Card Games

In total, 12% (6/49) of the systems use classical card games. In Blackjack (Sys1) [50], players strive to assemble a hand, achieving a value as close to 21 as feasible without exceeding this pivotal threshold. This game demands decision-making, blending risk assessment and strategic calculation elements. Free Cell (Sys1) [48] and Solitaire (Sys17) [80-83] epitomize the genre of sorting card games, necessitating the arrangement of a standard 52-card deck based on criteria of suits and ranks. These games not only require sequencing and strategic planning but also underscore the virtues of patience and foresight. The Find the Pair (Sys9, Sys15, and Sys48) [69,78,127] game challenges participants to unveil matching pairs from an array of face-down cards. Participation in this game demands memory and concentration. Finally, War (Sys26) [99] is a card-based contest where players select cards to show a higher rank against a computer-simulated adversary.

#### Digital Versions of Analog Games

Similar to card games, digitized versions of traditional board games remain popular and are used in GISs. Scrabble (Sys1 and Sys5) [49,57,58] is a popular word game where players use letter tiles to create words on a game board. The goal is to score the most points by forming words on a 15×15 grid board. Sudoku (Sys5) [57,58] is a logic-based number puzzle game. The objective is to fill a 9×9 grid with numbers so that each column, row, and each of the nine 3×3 subgrids (also known as “regions” or “boxes”) contains all the digits from 1 to 9 without repeating any numbers. Quick, Draw! (Sys34) [111] is a fun and fast-paced drawing game where players try to quickly draw an object within 20 seconds for artificial intelligence (AI) to guess.

#### Commercial Video Games

Besides traditional card and analog games, adaptations of classic video games were also used to assess cognitive functions. Pac-Man (Sys37) [115] is a game where the player controls Pac-Man, a yellow, circular character, navigating a maze while eating pellets and avoiding ghosts. In Tetris (Sys39) [118], the player controls falling tetrominoes, geometric shapes made up of 4 squares each, and aims to complete horizontal lines without gaps. Fruit Ninja (Sys39) [118] is a mobile game where the player slices various fruits that appear on the screen while avoiding bombs. Candy Crush Saga (Sys39) [118] is a popular match-3 puzzle game where players swap adjacent candies to form lines of ≥3 matching candies. Minecraft (Sys41) [120] is a sandbox video game that allows players to explore, build, and create in a blocky, procedurally generated 3D world.

#### Minigames

Several systems (21/49, 43%) were designed with simple gameplay to provide a fun game experience to older adults. Whack-a-Mole (Sys11, Sys28, and Sys29) [72,101,102] is originally an action-reaction game in which players hit moles that randomly pop up from holes on a game surface. The narrative of the game can also involve taking photos of animals (Sys38) [116,117] or beating devils (Sys48) [127]. Spot the Difference (Sys15) [78] is a puzzle game in which 2 images that appear similar are presented side by side but with subtle differences between them. The player’s objective is to carefully examine both images and identify discrepancies, such as missing objects, changed colors, or altered shapes, within a set time limit. Bounce Balls (Sys46) [125] is a game to bounce balls to break or eliminate objects on top of the screen. Counting animals (Sys19 and Sys21) [85,86,93,94] is a game where sheep move from one side of the screen to another within a certain time. Occasionally, wolves accompany the sheep. Players must answer questions related to the number of sheep or wolves. Lucy (Sys33) [109,110] gamified the cognitive tests with dots on the screen. Participants use a controller to select the right dots based on task requirements. Similarly, X-trop (Sys49) [128] gamified the trail making test (TMT) in a sailing environment. Other games without a specific name were also identified in the studies, such as *filling in the missing part of words* (Sys38) [116,117], *pairing words* with similar or opposite meanings (Sys14), *memorizing the location of mines* in a grid (Sys14) [76,77], *jigsaw* puzzles (Sys14 and Sys45) [76,77,124], *quizzes* (Sys16, Sys31, Sys 32, and Sys36) [79,107,108,113,114], and *moving the avatar to receive falling apples* (Sys35) [112].

#### Simulation Games

#### Overview

Daily living activities are typically not significantly impaired for older adults. Nonetheless, GISs have the potential to identify the deterioration of cognitive abilities in a controlled setting. Simulation games (17/49, 35%) replicate virtual 3D environments for older adults, usually their regular living environment. Here, as for the interaction paradigm, extended reality technology is often adopted for a more immersive experience for older adults (eg, virtual reality headsets), but some also still run on more traditional platforms that can be operated with a touch screen or a keyboard and a computer mouse. In these games, older adults are required to complete tasks, and the GISs can store their behavior and performance to track their progress. We break simulation games further down based on the virtual environment provided.

#### Shopping

Shopping is a crucial daily activity for older adults as it necessitates the use of multiple cognitive functions to maintain independence. Our research identified 7 different GISs that contain shopping experiences [53,54,74,96-98,103-106,126]. Shopping simulation has 2 main tasks: finding items and making payments. Some systems show the shopping list on the screen (Sys3, Sys24, Sys25, Sys30, and Sys47) [53,54,97,98,103,126], and some systems require players to memorize the shopping list beforehand (Sys12 and Sys23) [74,96].

#### Wayfinding

Wayfinding is another important daily activity for older adults. We found 6 GISs that simulate pathfinding. These systems simulate various environments for older adults, including a park (Sys4) [55,56], a museum (Sys10) [70], and driving (Sys40) [119] or walking (Sys47) [126] on city roads. Furthermore, there are also 2 systems that offer gamified tests to find the proper bus (Sys22) [95] or metro line (Sys38) [116,117].

#### Other “Activities of Daily Life”

Four systems simulate cooking (Sys8, Sys12, Sys23, and Sys44) [68,74,96,123]. Older adults find food and ingredients in the kitchen and then plan the steps of cooking. Two systems simulated the financing on the ATM machine (Sys22 and Sys25) [95,98]. Older adults should insert a card, choose the amount and type of notes, enter the password to print the receipt, and then retrieve the card. Two systems require older adults to remember a phone number and then dial it (Sys6 and Sys25) [59-64,98]. Six systems require older adults to identify objects or people. In SmartAging (Sys6) [59-64], older adults are instructed to locate a list of items in a kitchen and then recall them later during another task. Virtual Action Planning Museum (Sys10) [70] presents objects visually and audibly in a virtual tour and prompts older adults to recall them. Sys23 [96] requires players to remember the medications they need to take, and then, they should pick the right pills and amounts from the bottles. RE@CH (Sys25) [98] has a task that requires players to recognize famous people, numbers, and advertisements. Researchers at Microsoft used the Hololens in Sys27 [100] to create an augmented reality environment for older adults. The older adults are then asked to identify “unnaturally placed objects” and correctly position them. CogWorldTravel (Sys38) [116,117] tests that older adults are able to differentiate between faces that have not previously appeared in the game. Panoramix (Sys20) [87-92] evaluates memorizing objects during a walk in the community, with the same mechanism as the California Verbal Learning Test.

#### Full-Body Movement Games

Four systems use novel full-body movement interaction paradigms to assess cognitive functions. FitForAll (Sys7) [65-67] assesses the cognitive functions of older adults through movement and performance in an exergame originally designed for rehabilitation. Sys18 [84] and Sys45 [124] developed games that combine body movement and quizzes using Kinect. Seas the Day (Sys42) [121] allows older adults to engage in enjoyable activities such as fishing, practicing tai chi, and rowing within a virtual reality environment.

#### Dedicated Interactive Games

Three systems created dedicated hardware to provide more interactive and tangible game experiences. Fun Cube (Sys2) [51,52] has created dedicated tangible devices comprising 6 cubes with mini screens. They use these devices to gamify the MoCA in their system. SmartKuber (Sys13) [75] had a similar idea of using tangible cubes in the gameplay. Unlike Fun Cube, the researchers behind SmartKuber integrated augmented reality technology with physical cubes. Older adults use a tablet’s camera to scan the cubes and play the game. Researchers involved in the development of Sys43 [122] were exploring the use of haptic and olfactory sensors to help older adults identify objects.

In conclusion to RQ1, a diverse array of game genres and interaction types have been used for the assessment of MCI. The breakdown of game types includes casual games (30/49, 61%), simulations (17/49, 35%), full-body movement games (4/49, 8%), and those with dedicated interaction (3/49, 6%). A tabular summary of GISs mapped to the game types is provided in Multimedia Appendix 3.

### RQ2: Which Cognitive Functions Are Assessed by GISs?

We examined the cognitive functions assessed by GISs based on the 6 cognitive functions evaluated in MoCA (*short-term memory*; *visuospatial abilities*; *executive functions*; *attention, concentration, and working memory*; *language*; and *orientation to time and place*).

#### Short-Term Memory

A total of 28 systems assessed older adults’ short-term memory in a manner similar to the MoCA. These assessments typically involve presenting information and then asking the older adults to recall it. Often, additional context is provided, such as memorizing a shopping list (Sys12 and Sys23) [74,96], recalling objects encountered during a walk (Sys20 and Sys47) [91,126], arranging dishes on a table (Sys 12) [74], or locating a list of items in a kitchen (Sys44) [123]. Other activities may include memorizing a 2D “mines field” (Sys14) [76,77] or playing Find the Pair card games (Sys9) [69]. A time limit is often involved in assessing memory, such as when participants are asked to remember the number of sheep that come and go on a farm (Sys19 and Sys21) [85,86,93,94].

#### Visuospatial Ability

In total, 21 systems evaluated visuospatial ability. The MoCA assesses visuospatial ability through tasks such as drawing a clock and a cube. GISs can make drawing enjoyable, such as by allowing AI to guess what you are drawing (Sys34) [111] or sketch museum artifacts (Sys32) [108]. The gameplay of games such as Tetris (Sys39) [118] or filling space with shapes in Tetris (Sys38) [116,117] also helps evaluate visuospatial ability. GISs also enable a realistic assessment of visuospatial ability through their graphics. Older adults can navigate a virtual city or park and complete tasks that are often combined with memory, such as finding their way back (Sys40) [119].

#### Executive Functions

A total of 23 systems evaluated executive functions. The TMT part B is used in the MoCA to evaluate executive functions. Some systems have implemented minigames or tests similar to TMT (Sys2) [51,52] or directly gamified TMT (Sys49) [128]. Card games such as FreeCell (Sys1) [48] and Solitaire (Sys17) [82] also require executive functions in planning the strategy for sorting suits. More realistic tasks, such as cooking with a recipe (Sys8, Sys12, Sys23, and Sys44) [68,74,96,122] and sorting objects by types and then placing them in the proper places in simulation games (Sys27) [100], can also evaluate executive functions.

#### Attention, Concentration, and Working Memory

In total, 25 systems assessed attention, concentration, and working memory. In Whack-a-Mole and its variants (Sys11, Sys28, Sys29, Sys38, and Sys48) [72,101,102,116,117,127], players must pay attention and concentrate on hitting the moles while avoiding distractors such as bombs. Games such as Pac-Man (Sys37) [115] and Fruit Ninja (Sys39) [118] naturally challenge attention to avoid faults and obtain higher scores. Other games, such as Find the Pair (Sys9) [69] and Sudoku (Sys5) [57,58], require working memory to temporarily memorize useful information, such as patterns on cards and missing numbers in rows or columns.

#### Language

A total of 10 systems evaluated language skills. Quiz games (Sys15, Sys16, and Sys36) [78,79,113,114] often feature tasks such as naming objects and spelling words that start with specific letters. In simulation games, naming objects can also be easily integrated into the gameplay. Scrabble (Sys1 and Sys5) [49,57,58] also provides an enjoyable approach to test language comprehension. Contexts such as text passwords of luggage locks (Sys38) [116,117] can also assess language understanding. However, the articles included in this review indicate that none of the evaluated systems require voice input, meaning that language fluency is not assessed by any of them.

#### Orientation to Time and Place

In total, 7 systems evaluated orientation. We could not find game mechanisms to test orientation to time naturally. Time orientation is tested in a manner similar to word comprehension. For example, use the password hint as today’s date (Sys22) [95] or directly ask it (Sys2) [51,52]. Typically, orientation to place was assessed through gamified tasks. The orientation of place is often evaluated by combining it with visuospatial abilities, specifically the ability to navigate a path in a virtual environment (Sys4, Sys10, Sys12, Sys31, Sys40, and Sys47) [55,56,70,74,107,119,126]. Older adults must first identify the position of avatars using their orientation skills and then integrate these skills with their visuospatial abilities to find their way.

In summary, memory (28/49, 57%), visuospatial abilities (21/49, 43%), executive functions (23/49, 47%), and attention (25/49, 51%) are assessed more frequently than language (10/49, 20%) and orientation (7/49, 14%). A few systems (6/49, 12%) integrate the assessment of all MoCA functions into their gameplay. A tabular summary of GISs mapped to cognitive functions is provided in Multimedia Appendix 4.

### RQ3: How Are GISs Evaluated (eg, Longitudinal or Cross-Sectional), and How Reliable Are These Studies in Reporting Diagnostic Performance?

#### Overview

The third RQ focused on evaluating the effectiveness of GISs. We first examined the research designs used in clinical validation studies to address this RQ. Next, we assessed the quality of these research designs and evaluated the performance in assessing MCI. Therefore, we excluded 47 articles in 26 systems (Sys1, Sys5, Sys8, Sys11, Sys12, Sys14, Sys15, Sys18, Sys19, Sys23, Sys24, Sys27, Sys31, Sys32, Sys34, Sys35, Sys37-47, and Sys49) that lacked clinical validation studies (longitudinal study or cross-sectional study). In addition, we omitted the study that relied on synthetic data in which AI agents simulated the performance of healthy older adults and patients with MCI (Sys26) [99]. The studies without clear reference standards were also excluded (Sys4 and Sys22) [55,95]. This left us with 31 studies comprising 20 systems that had validation studies specifically aimed at assessing MCI. Finally, we summarized and reported the diagnostic performance of systems with clinical validation studies.

#### How Are GISs Evaluated?

Two studies (Sys13 and Sys14) [75,77] reported intraperson comparisons across sessions. Sys14 [77] involved 9000 healthy participants from different age groups playing 100 games while data were collected. The results showed a linear trend (*P*<.001) between scores and the number of sessions played, demonstrating that all age groups showed improvement in performance across all minigames aimed at assessing cognitive functions related to MCI. While performance improved for all age groups, older adults showed slower progress compared to younger participants. Sys13 [75] compared the mean total score of the last 20% of sessions with the scores from the first 20% of sessions in a 6-week study with 13 participants, where participants were asked to play the game twice a week. The results revealed that there was no statistically significant difference.

The remaining 29 studies on 18 systems presented a cross-sectional design. Validation methods included (1) statistical analysis of game metrics to differentiate individuals with MCI from healthy controls and (2) machine learning classification methods. These studies are discussed in more detail in the following sections.

#### How Reliable Are These Studies in Assessing Accuracy?

The included studies were evaluated on 3 dimensions (reference standard, relevancy of controls, and sample size), each of which is scored from 1 to 3, with a higher score indicating a higher quality, based on the quality assessment method adapted from other studies [45-47]. Note that the evaluation targets the quality of task classification in healthy older adults with MCI, not the overall study quality for different research goals.

#### Reference Standard

Studies in the review had varying criteria for enrolling participants as patients with MCI or healthy controls. The gold standard for diagnosing MCI is a psychophysiological examination conducted by health care professionals. A total of 14 studies explicitly stated that participants with MCI underwent a comprehensive clinical diagnosis (Sys3, Sys6, Sys7, Sys9, Sys10, Sys17, Sys20, Sys30, Sys33, and Sys48) [54,63,64,67,69,70,82,83,91,104-106,109,127]. Five studies used test batteries created by authors as reference standards (Sys4 and Sys20) [56,88-90,92]. In total, 10 studies used MMSE or MoCA as their reference standard (Sys2, Sys6, Sys16, Sys21, Sys25, Sys28, Sys29, and Sys36) [52,62,79,93,94,98,101, 102,113,114].

#### Relevance of Controls

In most studies, healthy controls were of comparable age to other groups (of patients with MCI). In 1 study [105] involving Sys30, the control group was older adults with subjective cognitive decline who visited clinics. These participants self-reported cognitive decline before cognitive tests could assess the deficits. However, subjective cognitive decline may be a precursor of nonnormative cognitive decline and eventual progression to MCI and dementia [130]. In addition to including older adults with MCI, a study [76] involving Sys14 used healthy young people as controls for control experiments. Given the natural decline in cognitive function with age, young adults may be more easily discriminated against than older adults.

The participant groups also varied across studies in their MCI subtypes. Some studies (Sys6, Sys10, Sys30, and Sys35) [63,64,70,104,112] recruited participants with amnestic MCI, while others did not differentiate between the MCI subtypes. Some studies (Sys6, Sys7, Sys8, Sys16, Sys20, Sys25, Sys26, Sys29, Sys33, Sys46, Sys48, and Sys49) [68,76,88,89,91,98,109,127] included patients with moderate to severe AD for comparison with both the group with MCI and healthy controls.

#### Sample Size

The average number of patients with MCI and healthy controls in the included studies was 27.44 (SD 18.63) and 42.75 (SD 51.79), respectively. Among all cross-sectional studies included in the review, 1 study on Sys6 [62] had the largest number of participants, 1086 in total. However, the reference standard was based on the MoCA administered by software. Sys48 [127] had the second largest sample size, with 240 in total (75 healthy controls, 41 patients with MCI, and 124patients with AD). Sys2 [52] had the largest MCI group sample size, with 65 participants. The study on Sys35 [112] had the largest number of healthy controls, totaling 280.

#### Quality of Studies

On the basis of the aforementioned analysis, the first author (YC) calculated study quality scores as defined in the Data Extraction subsection under the Methods section. There were 9 low-quality studies on 7 systems (Sys6, Sys13, Sys14, Sys20, Sys21, Sys28, and Sys36), 8 medium-quality studies on 8 systems (Sys2, Sys3, Sys4, Sys9, Sys16, Sys20, Sys25, and Sys29), and 14 high-quality studies on 8 systems (Sys6, Sys7, Sys10, Sys17, Sys20, Sys30, Sys33, and Sys48). Detailed scores are shown in Table 2.

**Table 2:** Quality of studies with respect to how reliable they are in assessing accuracy (3-5 indicates low quality, 6-7 indicates moderate quality, and 8-9 indicates high quality).

System number	Name of the gamified interactive system	Reference	Reference standard	Relevance of controls	Sample size	Quality of studies
Sys2	Fun Cube	[52]	1	2	3	6
Sys3	VAP-S^a^	[54]	3	2	2	7
Sys4	VREAD	[56]	2	2	3	7
Sys6	SmartAging	[62]	1	1	3	5
Sys6	SmartAging	[63]	3	3	3	9
Sys6	SmartAging	[64]	3	2	3	8
Sys7	FitForAll	[67]	3	3	3	9
Sys9	Find the pair	[69]	3	1	3	7
Sys10	VAP-M^b^	[70]	3	2	3	8
Sys13	Smartkuber	[75]	1	1	1	3
Sys14	—^c^	[77]	1	1	3	5
Sys16	—	[79]	1	2	3	6
Sys17	Dr. Solitaire	[82]	3	2	3	8
Sys17	Dr. Solitaire	[83]	3	2	3	8
Sys20	Panoramix	[88]	2	2	1	5
Sys20	Panoramix	[89]	2	3	2	7
Sys20	Panoramix	[90]	2	3	3	8
Sys20	Panoramix	[91]	2	3	3	8
Sys20	Panoramix	[92]	3	3	2	8
Sys21	Neuro-World	[93]	1	1	2	4
Sys21	Neuro-World	[94]	1	1	2	4
Sys25	RE@CH	[98]	1	2	3	6
Sys28	Holey Moley	[101]	1	1	3	5
Sys29	Hit-the-ball	[102]	1	3	3	7
Sys30	Virtual Supermarket	[104]	3	2	3	8
Sys30	Virtual Supermarket	[105]	3	2	3	8
Sys30	Virtual Supermarket	[106]	3	3	3	9
Sys33	Lucy	[109]	3	2	3	8
Sys36	COGNIPLAT	[113]	1	2	1	4
Sys36	COGNIPLAT	[114]	1	2	1	4
Sys48	The Ryokansan	[127]	3	3	3	9

^a^AUC: area under the curve.

^b^VAP-S: virtual action planning supermarket.

^c^Not available.

^d^HRMG: high-resolution monitoring games.

^e^MoCA: Montreal Cognitive Assessment.

^f^MMSE: Mini-Mental State Examination.

^g^EEG: electroencephalogram.

#### What Diagnostic Performances Are Reported?

In total, 19 studies comprising 14 different systems reported accuracy. The diagnostic performances are summarized in Table 3. The performance of GISs in assessing MCI shows promising results, with sensitivity ranging from 70.7% to 100%. This is comparable to MoCA, which has a sensitivity of 90% and is significantly better than the MMSE, which has a much lower sensitivity of 18% [20]. The specificity ranges from 56.5% to 100%. The best-performed GISs were also comparable with MMSE (94.7%) [131] and MoCA (87%) [132]. The AUC of the included GISs ranged from 0.774 to 1, which is also comparable with MMSE (78%) [43] and MoCA (83.3%) [43]. The following paragraphs discuss studies that demonstrated performance exceeding that of the MoCA and MMSE.

Among all 14 systems, the GISs with the best performance (Sys3) [54] demonstrated a sensitivity of 100%, a specificity of 100%, and an AUC of 1 in the task of classifying 2 groups (healthy older adults versus older adults with MCI or AD). However, the study was a single-center, nonblinded, cross-sectional study and had 6 healthy older adults and 6 older adults with MCI or early AD, 12 participants in total. Participants were recruited from 1 hospital.

The best-performing model for Panoramix (Sys20) [89] was the random forest. It achieved a sensitivity of 100%, a specificity of 70%, and an AUC of 0.98 for the task of classifying MCI among 3 groups (healthy older adults, older adults with MCI, and older adults with AD). The study included 64 participants: 28 healthy older adults, 16 older adults with MCI, and 20 older adults with AD. The newer version, Panoramix (version 2.0; Sys20) [91], also demonstrated a sensitivity of 100% and a specificity of 100% for the same task in a sample size of 30 participants, containing 10 healthy older adults, 10 older adults with MCI, and 10 older adults with AD. Notably, both Panoramix studies were single-center, nonblinded, cross-sectional studies. Participants were recruited from 1 association.

The study on Smart Aging (Sys6) [63] achieved an AUC of 0.986 (95% CI 0.962-1.000; *P*<.001) in distinguishing healthy older adults from those with cognitive impairment. The study was a single-center, nonblinded, cross-sectional investigation involving 91 participants: 23 older adults with amnestic MCI, 20 older adults with Parkinson disease–related MCI, and 25 older adults with AD.

The study on Holey Moley (Sys28) [101] had a sensitivity of 100%, a specificity of 97.2%, and an AUC of 0.953 in classifying cognitively healthy older adults and those with cognitive impairment. The study was a nonblinded, cross-sectional investigation involving 47 participants recruited from a single neurology clinic. Cognitive impairment was identified using the MMSE as the reference standard. However, the study did not report the specific cutoff score used or distinguish between MCI and AD. It only stated that 12 out of 47 participants were classified as cognitively impaired.

In total, 2 studies were conducted on COGNIPLAT (Sys36) [113,114]. Both studies were single-center, nonblinded, cross-sectional studies. The reference standard for cognitive impairment was MoCA, corrected by education level. In 1 study [113], researchers collected 119 game sessions from 10 participants. The best-performing model was a support vector machine with 9 variables from the game as input. The model had a sensitivity of 91.8% and a specificity of 93.2%. In the other study [114], researchers collected 119 game sessions from 10 participants. The best-performing model was the multilayer perceptron, with a sensitivity of 96.6% and a specificity of 90%. The model training variables included demographic data (eg, age and education) and game data (eg, game time and points).

**Table 3:** Mild cognitive impairment diagnosis performance.

System number	Name of the gamified interactive system	Reference	Game metrics	Sensitivity (%)	Specificity (%)	AUC^a^
Sys3	VAP-S^b^	[54]	Features extracted from trajectory, region, and task performance	100	100	1
Sys4	VREAD	[55]	Correct path, incorrect path, correct sequences, incorrect sequences, overall score, and time	75	96	—^c^
Sys6	SmartAging	[63]	Smart Aging Total Score	—	—	0.99
Sys6	SmartAging	[64]	Smart Aging Serious Game total score	84.4	75.5	0.88
Sys7	FitForAll	[67]	Evaluator-ranked age, HRMG^d^ mean total, HRMG intercept total, HRMG mean level 1, HRMG mean level3, HRMG mean level 4, and heart rate slope level 3	—	—	0.77
Sys9	Find the pair	[69]	Game trials	83	62	—
Sys9	Find the pair	[69]	Game time	82	67	—^c^
Sys16	—	[79]	Best cutoff game points based on MoCA^e^	88	77.8	—^c^
Sys16	—	[79]	Best cutoff game points based on MoCA	87.5	33.3	—^c^
Sys17	Dr. Solitaire	[83]	Nu-support vector classifier using digital biomarkers of cognitive performance in Klondike Solitaire	77.8	88.9	0.9
Sys20	Panoramix	[90]	Random forest using Panoramix battery	100	100	0.98
Sys20	Panoramix	[91]	Support vector machine using Panoramix (version 2.0) battery	100	100	—
Sys25	RE@CH	[98]	Total performance score	78.2	75.7	0.82
Sys28	Holey Moley	[101]	Holey Moley game classification based on MMSE^f^	100	97.2	0.95
Sys30	Virtual Supermarket	[104]	Score in the Virtual Supermarket Program	85.9	79	0.87
Sys30	Virtual Supermarket	[105]	All Virtual Supermarket variables	76.3	91.4	—
Sys30	Virtual Supermarket	[106]	All Virtual Supermarket variables	74	85	—
Sys30	Virtual Supermarket	[106]	All bought unlisted, correct money, and duration	79	86	—
Sys33	Lucy	[109]	Focused attention response profile and EEG^g^ characteristics	80	82.6	—
Sys36	COGNIPLAT	[113]	Support vector machine using 9 variables	91.8	93.2	—
Sys36	COGNIPLAT	[114]	Manually selected feature set with 9 features: age, family medical history, exercising, education, average game round time in game session, orientation game importance, naming game importance, memory game importance, and recall (Anakilisi) game importance	96.6	90	0.99
Sys48	The Ryokansan	[127]	Flipping cards game score	76.9	70.7	—
Sys48	The Ryokansan	[127]	Game score from the “finding mistakes” task	70.7	56.5	—

^a^AUC: area under the curve.

^b^VAP-S: virtual action planning supermarket.

^c^Not available.

^d^HRMG: high-resolution monitoring games.

^e^MoCA: Montreal Cognitive Assessment.

^f^MMSE: Mini-Mental State Examination.

^g^EEG: electroencephalogram.

## Discussion

### Principal Findings

We identified 49 GISs for assessing MCI in this scoping review, mostly casual games and simulations, that support engagement through simple game mechanics or familiarity with daily tasks, such as shopping. We further identified that GISs mainly targeted cognitive functions, such as memory, attention, and executive function, but often lacked depth in language and orientation assessment. Finally, we found that only 14 systems reported diagnostic performance, with other studies remaining in early design stages or offering limited correlation studies. Moreover, although several GISs reported strong diagnostic performances, many of these studies (9/31, 29%) were limited by small samples, methodological flaws, and overfitting risks. In the following sections, we revisit our 3 RQs in more depth.

### RQ1: What Are the Different Game Types (Genres and Interaction Paradigms) of GISs Used to Assess MCI?

We found that most GISs (30/49, 61%) consist of casual games with clear rules and straightforward gameplay, making them accessible and easy to play without requiring extensive learning or specialized technology. This simplicity may encourage older adults to engage with these systems, addressing the challenge that older adults generally have a lower game participation rate than other age groups [133]. We also noted the popularity of simulations (17/49, 35%). These offer a unique opportunity to represent and assess daily activities in a more controlled environment, providing valuable supportive data with higher ecological validity for evaluating MCI. In particular, shopping experiences are commonly integrated into many simulation game systems (7/49, 14%). The shopping experience is indeed a significant daily activity that requires short-term memory to memorize the shopping list, executive functions to recognize items, and spatial orientation to find them. Only a few systems (4/49, 8%) use full-body movement interaction paradigms to assess MCI. These include exergames and Kinect-based quiz games. Finally, only a few systems (3/49, 6%) were developed with dedicated hardware for the assessment of MCI, representing an extension of traditional GISs design.

In addition, we identified shared characteristics among the games examined in this study. All the GISs included in this research were noncompetitive and offered a single-player mode. Each gaming session was relatively brief, lasting around 15 minutes, and designed to be easy to understand. These observations align with the preference of older adults for puzzles and intellectually stimulating games [134]. Nevertheless, we like to emphasize the ongoing debate regarding whether merely simulation experiences can genuinely be considered games [135]. When daily activities are simulated without a well-integrated game design, it may lead to a lackluster gaming experience [136,137]. Furthermore, the gaming experience of casual games may not provide *meaningful* play for all older audiences. As articulated in the *gerontoludic manifesto* [138], game and mental health researchers may want to put more effort into understanding what differentiates older players rather than considering them as united in their age-related impairments.

Although we categorized gamified systems into *casual*, *simulation*, *full-body movement*, or *dedicated interactive* games for descriptive purposes, it is important to note that these genre distinctions are blurry in practice. Many games combine features from multiple genres, and genre labels do not necessarily reflect the specific cognitive demands or mechanisms embedded within gameplay. In addition, most studies did not describe game mechanics in sufficient detail to allow for systematic mapping to targeted cognitive functions. This limits the extent to which we can draw mechanistic conclusions about genre-specific diagnostic performance.

### RQ2: Which Cognitive Functions Are Assessed by GISs?

The scoping review revealed that a diverse range of cognitive functions were assessed by GISs, including short-term memory (28/49, 57% GISs), visuospatial skills (20/49, 41% GISs), executive functions (23/49, 47% GISs), and attention and working memory (25/49, 51% GISs). However, a significant gap exists in evaluating language abilities (10/49, 20% GISs) and orientation to time and space (7/49, 14% GISs).

Moreover, the GISs reviewed in this study vary in how comprehensively they address the 6 cognitive domains evaluated by the MoCA (ie, short-term memory, visuospatial abilities, executive functions, attention, language, and orientation). For example, some simulation games incorporate daily tasks such as shopping, cooking, or wayfinding that naturally engage multiple domains, particularly short-term memory, executive functions, and visuospatial abilities. In contrast, casual games more often target specific domains, such as attention or working memory, through task-focused mechanics such as matching, sequencing, or quick-response challenges. Full-body movement games and dedicated interactive systems were fewer in number, and their alignment with MoCA domains varied by design, with some addressing attention or memory through physical interaction.

It is important to note that many GISs incorporate features from multiple game types, and genre labels do not consistently reflect the underlying cognitive functions. In addition, we could not draw definitive conclusions about the relative diagnostic effectiveness of different game types due to heterogeneity in the reporting of GISs designs. A better understanding of how specific game elements engage specific cognitive functions can guide future design and evaluation efforts. Hence, a more granular analysis of game mechanics in relation to targeted cognitive functions—beyond genre—would strengthen future investigations.

In particular, GISs could extend the duration of their assessment. The engaging nature of GISs can potentially encourage older adults to use the system for extended periods, creating opportunities to collect data over a longer time, in a manner that might not be feasible through traditional assessment tools. Thus, GISs could play a valuable role in evaluating long-term cognitive functions by tracking subtle changes over time, providing extra information that current screening instruments, such as MMSE and MoCA, are not designed to capture. Despite this potential, no existing system was identified in this review that specifically targets long-term MCI evaluation. Hence, future studies should prioritize the development and validation of GISs that leverage game mechanics to promote repeated use and sustained engagement, while also exploring how these systems can complement traditional diagnostic methods.

### RQ3: How Are GISs Evaluated (eg, Longitudinal or Cross-Sectional), and How Reliable Are These Studies in Reporting Diagnostic Performance?

Out of a total of 49 GISs, 29 (59%) remain in the system design stage or present a preliminary user study. Hence, these GISs lack evidence of accuracy. Moreover, many studies (6/49, 12%) conducted on GIS were limited to correlating system metrics with traditional screening instruments, reducing their scientific rigor. In total, only 29% (14/49) of the GISs reported clinical validation to classify patients with MCI or intraperson comparisons.

The quality scores presented in Table 2 highlight notable variations in the methodological rigor of studies evaluating GISs for MCI assessment. While some studies used well-defined reference standards, relatively larger sample sizes, and appropriate validation strategies, others lacked clarity in participant selection or relied on less robust evaluation methods. This variability should be considered when interpreting reported diagnostic performance; higher-quality studies are more likely to yield reliable and generalizable findings, yet the results may be more nuanced. For researchers and clinicians, these scores offer a practical reference point for assessing the strength of evidence and identifying areas where future studies can improve in terms of design transparency and reporting consistency.

While clinical validation studies indicate that GISs may be a useful tool for assessing MCI, in particular, the risk of model overfitting and methodological bias remains a critical concern. For example, the virtual action planning supermarket model achieved an extraordinary F_1_-score of 1.0 across 4 machine learning algorithms: decision trees, random forests, adaptive boosting, and extreme gradient boosting. Such a result must be interpreted with extreme caution. The study included only 12 participants and used data enhancement techniques, yet identified 45 out of 46 features as significant—without any dimensionality reduction. Such a high feature-to-sample ratio introduces a substantial risk of overfitting, undermining the robustness of the findings.

More broadly, studies reporting near-perfect performance (eg, 1.0 accuracy or *F*_1_-score) are likely reflecting limitations in sample size, study design, or evaluation procedures rather than actual diagnostic capability. To prevent misinterpretation, especially by third-party stakeholders, it is essential to frame such results as preliminary and methodologically constrained results. These findings should not be taken as evidence of clinical readiness**.**

A robust study design is crucial for the clinical validation of GISs. First, the use of age-matched control groups is essential to minimize confounding variables. A key limitation in interpreting the performance of GISs-based cognitive assessments is the potential confounding effect of previous gaming experience or digital proficiency. Younger participants may outperform older adults not solely due to better cognitive functioning but also because of greater familiarity with digital devices and interaction patterns common in games. Only a few of the reviewed studies (4/31, 13%) assessed or controlled for previous gaming experience, making it difficult to disentangle cognitive performance from gameplay proficiency, as shown in the results for RQ3. This issue is further supported by recent findings from the study by Murata et al [139], which demonstrate that peripheral metrics such as swipe speed—independent of game content—can predict cognitive impairment. Future research should consider including baseline assessments of digital literacy or gaming familiarity and explore interface designs that minimize performance biases related to device handling rather than cognitive ability. In addition, previous research has highlighted sex-related differences in the risk, progression, and presentation of MCI [140,141]; most of the included studies (30/31, 97%) did not report results disaggregated by sex or analyze potential sex-based differences in diagnostic performance. As such, our review was unable to assess how sex may influence the effectiveness or generalizability of GISs for MCI assessment. The lack of such analyses represents a critical gap in the literature. Future research should explicitly report and analyze sex-disaggregated data to ensure that these systems are equitably designed and validated across diverse populations. Nevertheless, it is encouraging to see the inclusion of diverse groups, such as participants with AD, as MCI is regarded as a transitional phase between healthy aging and AD.

Second, comprehensive cognitive assessments should be administered to all participants, with those diagnosed with MCI specifically evaluated by health care professionals to ensure diagnostic precision. Solely relying on screening instruments for labeling MCI is inadequate and may overlook the complexity of cognitive decline.

Furthermore, most existing studies involve small, single-center cohorts, which limits generalizability. In addition, all the studies in the review used retrospective case-control designs, which can overestimate diagnostic performance by comparing well-defined cases and controls in artificial conditions. This introduces a high risk of bias and limits applicability in real-world diagnostic contexts. Future research would benefit from adopting larger, blinded, and multicenter studies—ideally through prospective diagnostic cohort designs—to strengthen the evidence base and enhance the clinical relevance of GISs.

Longitudinal studies, in particular, could facilitate a deeper understanding of long-term memory and its association with other cognitive functions, broadening the scope of cognitive domains assessed through GISs. By leveraging the engaging nature of gamified systems, researchers can explore how sustained interactions might reveal patterns or markers indicative of MCI progression or even early-stage cognitive decline. Future research could focus on designing and implementing longitudinal studies that incorporate diverse user populations, validate their findings against established clinical benchmarks, and investigate the potential of GISs to serve as an early warning system for cognitive decline. However, longitudinal studies present several challenges, such as participant retention, data standardization, and the ethical implications of prolonged data collection. Addressing these will be essential to maximize the utility and reliability of GISs in clinical practice.

### Limitations

This review has several limitations. First, we did not expand our search beyond the initially included articles, which may have resulted in the exclusion of relevant studies related to specific GISs. For instance, in some cases, we only captured validation studies, while related user studies could not be identified from our database search. Second, our understanding of games within GISs was primarily derived from text descriptions and screenshots in the articles, without any direct interaction with the systems themselves. Consequently, the findings from RQ1 and RQ2 rely on the authors’ interpretations rather than firsthand experiences. This reliance on secondary information may restrict the accuracy and completeness of our descriptions of GISs. The community has also noted the challenges in articulating artifacts in experimental game research, and efforts are currently underway to refine standards [142].

### Conclusions

GISs hold considerable potential to enhance the diagnostic process for MCI by capturing high-frequency, rich, and ecologically valid data, offering new insights beyond traditional screening instruments and test batteries. This rich data collection could provide health care professionals with a complementary perspective for diagnosis, enabling a more dynamic understanding of cognitive changes over time. To this end, this paper presents a scoping review of GISs used for assessing MCI. In total, 49 GISs were identified from 81 published articles and categorized into 4 types: casual games, simulation games, full-body movement games, and dedicated interactive games. We also examined the cognitive functions targeted and the accompanying diagnostic performance validation studies. Findings indicate a broad spectrum of GISs available for evaluating MCI with a wide range of cognitive functions. In addition, we identified several GISs reporting diagnostic performances comparable to screening instruments such as MMSE and MoCA—including a few studies (6/31, 19%) with near-perfect results. However, these findings should be interpreted with caution, as they often stem from studies with small sample sizes and limited methodological rigor. The analysis also identified limitations in the GISs validation; only 2 systems included in this review conducted intraperson comparison studies, and no rigorous longitudinal study was identified. This gap represents a missed opportunity to investigate the full potential of GISs in assessing MCI progression. Such studies could provide critical insights into how gameplay proficiency, learning effects, and adaptation to game mechanics influence the evaluation of cognitive functions, thereby enhancing the interpretability and accuracy of these GISs. Overall, this review underscores the significant potential of GISs in assessing MCI, while highlighting the need for continued research to yield more conclusive results across various facets of these interactive systems.

## Appendix

Multimedia Appendix 1 Protocol for a scoping review of video games and gamification for assessing mild cognitive impairment.

Multimedia Appendix 2 Search queries.

Multimedia Appendix 3 Systems and game genres included in the review.

Multimedia Appendix 4 Cognitive functions evaluated in systems.

Multimedia Appendix 5 PRISMA-ScR (Preferred Reporting Items for Systematic Reviews and Meta-Analyses extension for Scoping Reviews) checklist.

## Abbreviations

AD: Alzheimer disease
AI: artificial intelligence
AUC: area under the curve
GISs: gamified interactive systems
MCI: mild cognitive impairment
MMSE: Mini-Mental State Examination
MoCA: Montreal Cognitive Assessment
PRISMA-ScR: Preferred Reporting Items for Systematic Reviews and Meta-Analyses extension for Scoping Reviews
RQ: research question
TMT: trail making test
