# Supplementary material for: Video Games and Gamification for Assessing Mild Cognitive Impairment: Scoping Review
Source: JMIR Ment Health. 2025 Aug 5;12:e71304. doi: 10.2196/71304 (PMC12401070; doi:10.2196/71304)
Supplement: Multimedia Appendix 1 [file mental_v12i1e71304_app1.docx]

Protocol for A Scoping Review of Video games and gamification for Assessing Mild Cognitive Impairment

Authors: Yu Chen^1^, Kathrin Gerling^2^, Katrien Verbert^1^, Vero Vanden Abeele^1^

1. KU Leuven, Belgium
2. Karlsruhe Institute of Technology, Germany

# Introduction

## Background

Early assessment of mild cognitive impairment (MCI) in older adults is crucial, as it enables timely interventions and supports more effective decision-making related to care planning, lifestyle adjustments, and clinical monitoring [1,2]. MCI is a clinical condition that often precedes dementia and is characterized by cognitive decline greater than expected for age and education level, but without significant interference in daily functioning [5]. Identifying MCI early increases the chances of delaying or preventing further cognitive deterioration [3,4].

Traditional cognitive screening tools, such as the Montreal Cognitive Assessment (MoCA) or Mini-Mental State Examination (MMSE), have been widely used in clinical settings. However, they often face limitations in terms of ecological validity, accessibility, and participant engagement [6-8]. These limitations have prompted growing interest in gamified interactive systems (GIS), which integrate game mechanics with cognitive tasks to create more engaging, user-friendly, and potentially more sensitive methods for cognitive assessment.

## Objective

- Review the existing systems to understand the different game types (including genres and interaction paradigms) employed for assessment.
- Examined the cognitive functions targeted.
- Investigate the evidence for the performance of assessing MCI through GIS by looking at the quality of validation for these systems in assessing MCI and the diagnostic performance reported.

## Keywords

Mild cognitive impairment; Gamification; Video games; Cognitive assessment; Serious games

# Methods

This scoping review aims to comprehensively examine the literature on the use of video games and gamification for assessing MCI. The review adopts a broad inclusion strategy to capture diverse systems and employs a narrative synthesis approach to present the results.

### Inclusion Criteria

Studies were included if they met the following criteria:

1. Full-text, peer-reviewed articles published in English
2. Evaluating MCI as a component or the entirety of its function
3. Explicit inclusion of game or gamified elements in the paper

### Exclusion Criteria

Studies were excluded if they met any of the following:

1. Studies featuring games or gamified elements in non-electronic forms, such as pen-and-paper games.
2. Game or gamified systems are designed only for rehabilitation or training of specific cognitive functions or targeting dementia instead of MCI.
3. Papers that did not claim implementation
4. Book chapters that are inaccessible or retracted papers.

## Information source

The following databases were searched:

- Scopus (which includes PubMed, MEDLINE, and EMBASE)
- IEEE Xplore
- ACM Digital Library

These databases were selected to cover both medical/clinical and technical domains.

## Search strategy

For the population, the search focused on patients with either "Mild Cognitive Impairment" or "MCI," deliberately excluding dementia to maintain an emphasis on the pre-stage of dementia. Regarding the instrument used for assessment, the search included the terms "Gamif*," "Game," "Video game," or "Videogame." Additionally, the purpose of the instrument was specified as "assessment," with variations like "Evaluation," "Screen*," and "Diagnos*" included as synonyms in the queries. These terms were searched across metadata, titles, abstracts, keywords, and full text.

### IEEE:

(("Full Text & Metadata": "MCI" OR "Full Text & Metadata": "mild cognitive impairment") AND ("Full Text & Metadata": "video game" OR "Full Text & Metadata": "videogame" OR "Full Text & Metadata": "gamif*" OR "Full Text & Metadata": "game") AND ("Full Text & Metadata": "assess*" OR "Full Text & Metadata": "evaluat*" OR "Full Text & Metadata": "measur*" OR "Full Text & Metadata": "screen*" OR "Full Text & Metadata": "diagno*"))

### Scopus:

### ("Full Text .AND. Metadata":"mild cognitive impairment" OR "Full Text .AND. Metadata":"mci") AND ("Full Text .AND. Metadata":game OR "Full Text .AND. Metadata":gamif* OR "Full Text .AND. Metadata":videogame OR "Full Text .AND. Metadata":"video game") AND ("Full Text .AND. Metadata":assess* OR "Full Text .AND. Metadata":evaluat* OR ""Full Text .AND. Metadata":screen* OR "Full Text .AND. Metadata":measure* OR "Full Text .AND. Metadata":diagno*)

### ACM DL:

( ALL ( "mild cognitive impairment" ) OR ALL ( mci ) ) AND ( ALL ( game ) OR ALL ( videogame ) OR ALL ( gamif* ) ALL ( "video game" ) ) AND ( ALL ( assess* ) OR ALL ( evaluat* ) OR ALL ( screen* ) OR ALL ( measure* ) OR ALL ( diagno* ) )

## Study selection

The first author independently conducted the screening of titles, abstracts, and full-text articles against the eligibility criteria. Given the clearly defined criteria, no second reviewer was involved in the screening process. No automation tools were used in the study selection process.

## Data extraction

The first author extracts the data from the included papers and then discusses this with the last author. The codes are:

- Description of GIS based on genre (e.g., casual games or simulation games) and interaction paradigm
- Cognitive functions match to those in MoCA
- Evaluation study design
- Scientific validation methods
- Ground truth for MCI
- Participants (health controls, MCI patients, and AD patients)
- Diagnostic performance (Sensitivity, Specificity, AUC)

## **Data Synthesis**

Given the exploratory nature of this review, a **narrative synthesis** approach will be used to summarize and report the findings. Extracted data will be organized into thematic categories aligned with the review objectives, including:

- **Game types and interaction paradigms**: categorized by genre (e.g., casual, simulation) and input methods (e.g., touchscreen, motion-based).
- **Targeted cognitive functions**: mapped against the domains assessed by the Montreal Cognitive Assessment (MoCA), such as memory, attention, executive function, and visuospatial abilities.
- **Evaluation and validation characteristics**: including study design, sample characteristics (e.g., healthy controls, MCI, AD), and the type of ground truth used for MCI identification.
- **Diagnostic performance**: summarized using available metrics such as sensitivity, specificity, and area under the curve (AUC).

Descriptive statistics will be reported where applicable (e.g., number of systems per game type, number of studies targeting each cognitive domain). Tables and figures will be used to visually summarize key patterns and distributions.

No meta-analysis or quantitative synthesis will be conducted, as this scoping review aims to map the field rather than evaluate effect sizes or comparative effectiveness.

# **Results**

This project was initially designed as a systematic review and later adapted into a scoping review following journal recommendations and a reassessment of the study aims. Given the diversity in study designs, outcome measures, and the emerging nature of the field, a scoping review was deemed more appropriate to comprehensively map the existing literature on gamified interactive systems (GIS) for assessing mild cognitive impairment (MCI).

The study is **self-funded** and was **registered on the Open Science Framework (OSF)** with register number m2sar. The search strategy and execution were inherited from the earlier systematic review effort. Searches were conducted in July 2024 across three major databases: Scopus, IEEE Xplore, and the ACM Digital Library. These searches yielded a total of 3856 records.

The full scoping review follows the PRISMA-ScR guidelines [9] and includes a flow diagram to summarize the study selection process.

# **Discussion**

This scoping review seeks to map the landscape of gamified interactive systems (GIS) developed for assessing mild cognitive impairment (MCI), with a particular focus on game types, targeted cognitive functions, and diagnostic performance. By synthesizing the existing body of literature, the review aims to provide researchers and practitioners with a clearer understanding of current design strategies, validation approaches, and cognitive assessment coverage in GIS.

One of the key contributions of this review is to organize and categorize diverse GIS efforts, which vary greatly in terms of genre, interaction paradigms, cognitive targeting, and evaluation rigor. As the field continues to grow, the findings from this review may help guide future system design, validation strategies, and the integration of user experience and clinical accuracy in gamified assessments.

The transition from a systematic to a scoping review reflects the need to embrace the heterogeneity in this emerging field and to provide a more flexible framework for identifying gaps and trends. The expected outcomes will offer a comprehensive overview rather than comparative effectiveness, which is appropriate given the exploratory nature of this research area.

## **Ethics and Dissemination**

As this review involves the analysis of data from previously published studies, no ethical approval is required. The results of the scoping review will be disseminated through submission to a peer-reviewed journal and presentations at relevant academic and professional conferences in the domains of digital health, cognitive assessment, and human-computer interaction.

## **Conflicts of Interest**

There are no conflicts of interest in this study.

# References

1. Chandler MJ, Parks AC, Marsiske M, Rotblatt LJ, Smith GE. Everyday Impact of Cognitive Interventions in Mild Cognitive Impairment: a Systematic Review and Meta-Analysis. Neuropsychol Rev 2016 Sep;26(3):225–251. doi: 10.1007/s11065-016-9330-4
2. Miller DI, Taler V, Davidson PSR, Messier C. Measuring the impact of exercise on cognitive aging: methodological issues. Neurobiol Aging 2012 Mar;33(3):622.e29-622.e43. doi: 10.1016/j.neurobiolaging.2011.02.020
3. Pergher V, Schoenmakers B, Demaerel P, Tournoy J, Van Hulle MM. Differential Impact of Cognitive Impairment in MCI Patients: A Case-Based Report. Case Rep Neurol 2020 Jun 29;12(2):222–231. doi: 10.1159/000507977
4. Livingston G, Sommerlad A, Orgeta V, Costafreda SG, Huntley J, Ames D, Ballard C, Banerjee S, Burns A, Cohen-Mansfield J, Cooper C, Fox N, Gitlin LN, Howard R, Kales HC, Larson EB, Ritchie K, Rockwood K, Sampson EL, Samus Q, Schneider LS, Selbæk G, Teri L, Mukadam N. Dementia prevention, intervention, and care. The Lancet 2017 Dec;390(10113):2673–2734. doi: 10.1016/S0140-6736(17)31363-6
5. Petersen RC. Mild Cognitive Impairment. N Engl J Med 2011 Jun 9;364(23):2227–2234. doi: 10.1056/NEJMcp0910237
6. Aminisani N, alimi R, Javadpour A, Asghari-Jafarabadi M, Jourian M, Stephens C, Shamshirgaran M. Comparison between the accuracy of Montreal Cognitive Assessment and Mini-Mental State Examination in the detection of mild cognitive impairment. In Review; 2021 Jan. doi: 10.21203/rs.3.rs-136185/v1
7. Horton DK, Hynan LS, Lacritz LH, Rossetti HC, Weiner MF, Cullum CM. An Abbreviated Montreal Cognitive Assessment (MoCA) for Dementia Screening. Clin Neuropsychol 2015 May 19;29(4):413–425. doi: 10.1080/13854046.2015.1043349
8. Nasreddine ZS, Phillips NA, Bédirian V, Charbonneau S, Whitehead V, Collin I, Cummings JL, Chertkow H. The Montreal Cognitive Assessment, MoCA: A Brief Screening Tool For Mild Cognitive Impairment. J Am Geriatr Soc 2005 Apr;53(4):695–699. doi: 10.1111/j.1532-5415.2005.53221.x
9. Tricco, AC, Lillie, E, Zarin, W, O'Brien, KK, Colquhoun, H, Levac, D, Moher, D, Peters, MD, Horsley, T, Weeks, L, Hempel, S et al. PRISMA extension for scoping reviews (PRISMA-ScR): checklist and explanation. Ann Intern Med. 2018,169(7):467-473. doi: [10.7326/M18-0850](https://doi.org/10.7326/M18-0850)
