# Supplementary material for: Video Games and Gamification for Assessing Mild Cognitive Impairment: Scoping Review
Source: JMIR Ment Health. 2025 Aug 5;12:e71304. doi: 10.2196/71304 (PMC12401070; doi:10.2196/71304)
Supplement: Multimedia Appendix 2 [file mental_v12i1e71304_app2.docx]

# Search queries

For the population, the search focused on patients with either "Mild Cognitive Impairment" or "MCI," deliberately excluding dementia to maintain an emphasis on the pre-stage of dementia. Regarding the instrument used for assessment, the search included the terms "Gamif*," "Game," "Video game," or "Videogame." Additionally, the purpose of the instrument was specified as "assessment," with variations like "Evaluation," "Screen*," and "Diagnos*" included as synonyms in the queries. These terms were searched across metadata, titles, abstracts, keywords, and full text.

### IEEE:

(("Full Text & Metadata": "MCI" OR "Full Text & Metadata": "mild cognitive impairment") AND ("Full Text & Metadata": "video game" OR "Full Text & Metadata": "videogame" OR "Full Text & Metadata": "gamif*" OR "Full Text & Metadata": "game") AND ("Full Text & Metadata": "assess*" OR "Full Text & Metadata": "evaluat*" OR "Full Text & Metadata": "measur*" OR "Full Text & Metadata": "screen*" OR "Full Text & Metadata": "diagno*"))

### Scopus:

### ("Full Text .AND. Metadata":"mild cognitive impairment" OR "Full Text .AND. Metadata":"mci") AND ("Full Text .AND. Metadata":game OR "Full Text .AND. Metadata":gamif* OR "Full Text .AND. Metadata":videogame OR "Full Text .AND. Metadata":"video game") AND ("Full Text .AND. Metadata":assess* OR "Full Text .AND. Metadata":evaluat* OR ""Full Text .AND. Metadata":screen* OR "Full Text .AND. Metadata":measure* OR "Full Text .AND. Metadata":diagno*)

### ACM DL:

( ALL ( "mild cognitive impairment" ) OR ALL ( mci ) ) AND ( ALL ( game ) OR ALL ( videogame ) OR ALL ( gamif* ) ALL ( "video game" ) ) AND ( ALL ( assess* ) OR ALL ( evaluat* ) OR ALL ( screen* ) OR ALL ( measure* ) OR ALL ( diagno* ) )
