# Supplementary material for: Video Games and Gamification for Assessing Mild Cognitive Impairment: Scoping Review
Source: JMIR Ment Health. 2025 Aug 5;12:e71304. doi: 10.2196/71304 (PMC12401070; doi:10.2196/71304)
Supplement: Multimedia Appendix 3 [file mental_v12i1e71304_app3.docx]

Multimedia Appendix 3: Systems and game genres included in the review

| **System number** | **Casual games (n = 30)** | | | | **Simulation (n = 17)** | | | **Full-body movement (n = 4)** | **Dedicated interaction (n = 3)** |
| --- | --- | --- | --- | --- | --- | --- | --- | --- | --- |
|  | **Card games (n = 6)** | **Digital board game (n = 4)** | **Commercial video games (n = 4)** | **Minigames (n = 21)** | **Shopping (n = 7)** | **Wayfinding (n = 6)** | **Other activity of daily life (n = 11)** |  |  |
| Sys1 | • | • |  |  |  |  |  |  |  |
| Sys2 |  |  |  |  |  |  |  |  | • |
| Sys3 |  |  |  |  | • |  |  |  |  |
| Sys4 |  | • |  | • |  | • |  |  |  |
| Sys5 |  | • |  |  |  |  |  |  |  |
| Sys6 |  |  |  |  |  |  | • |  |  |
| Sys7 |  |  |  |  |  |  |  | • |  |
| Sys8 |  |  |  |  |  |  | • |  |  |
| Sys9 | • |  |  |  |  |  |  |  |  |
| Sys10 |  |  |  |  |  | • | • |  |  |
| Sys11 |  |  |  | • |  |  |  |  |  |
| Sys12 |  |  |  |  | • |  | • |  |  |
| Sys13 |  |  |  |  |  |  |  |  | • |
| Sys14 |  |  |  | • |  |  |  |  |  |
| Sys15 | • |  |  | • |  |  |  |  |  |
| Sys16 |  |  |  | • |  |  |  |  |  |
| Sys17 | • |  | • |  |  |  |  |  |  |
| Sys18 |  |  |  |  |  |  |  | • |  |
| Sys19 |  |  |  | • |  |  |  |  |  |
| Sys20 |  |  |  | • |  |  | • |  |  |
| Sys21 |  |  |  | • |  |  |  |  |  |
| Sys22 |  |  |  |  |  | • | • |  |  |
| Sys23 |  |  |  |  | • |  | • |  |  |
| Sys24 |  |  |  | • | • |  |  |  |  |
| Sys25 |  |  |  |  | • |  | • |  |  |
| Sys26 | • |  |  |  |  |  |  |  |  |
| Sys27 |  |  |  |  |  |  | • |  |  |
| Sys28 |  |  |  | • |  |  |  |  |  |
| Sys29 |  |  |  | • |  |  |  |  |  |
| Sys30 |  |  |  |  | • |  |  |  |  |
| Sys31 |  |  |  | • |  |  |  |  |  |
| Sys32 |  |  |  | • |  |  |  |  |  |
| Sys33 |  |  |  | • |  |  |  |  |  |
| Sys34 |  | • |  |  |  |  |  |  |  |
| Sys35 |  |  |  | • |  |  |  |  |  |
| Sys36 |  |  |  | • |  |  |  |  |  |
| Sys37 |  |  | • |  |  |  |  |  |  |
| Sys38 |  |  |  | • |  | • | • |  |  |
| Sys39 |  |  | • |  |  |  |  |  |  |
| Sys40 |  |  |  |  |  | • |  |  |  |
| Sys41 |  |  | • |  |  |  |  |  |  |
| Sys42 |  |  |  |  |  |  |  | • |  |
| Sys43 |  |  |  |  |  |  |  |  | • |
| Sys44 |  |  |  |  |  |  | • |  |  |
| Sys45 |  |  |  | • |  |  |  | • |  |
| Sys46 |  |  |  | • |  |  |  |  |  |
| Sys47 |  |  |  |  | • | • |  |  |  |
| Sys48 | • |  |  | • |  |  |  |  |  |
| Sys49 |  |  |  | • |  |  |  |  |  |
