# Supplementary material for: Video Games and Gamification for Assessing Mild Cognitive Impairment: Scoping Review
Source: JMIR Ment Health. 2025 Aug 5;12:e71304. doi: 10.2196/71304 (PMC12401070; doi:10.2196/71304)
Supplement: Multimedia Appendix 4 [file mental_v12i1e71304_app4.docx]

Multimedia Appendix 4: Cognitive functions evaluated in systems

| **System #** | **Name** | **Short-term memory (n = 28)** | **Visuospatial ability** **(n = 21)** | **Executive functions** **(n = 23)** | **Attention, Concentration, and working menory** **( n = 25)** | **Language** **(n = 10)** | **Orientation to time and place** **(n = 7)** |
| --- | --- | --- | --- | --- | --- | --- | --- |
| Sys1 |  |  |  | • | • |  |  |
| Sys2 | Fun Cube | • | • | • | • | • | • |
| Sys3 | VAP-S | • |  | • |  |  |  |
| Sys4 | VREAD | • | • |  |  |  |  |
| Sys5 | CWG\CSG |  |  |  |  |  |  |
| Sys6 | SmartAgeing | • | • | • | • | • | • |
| Sys7 | FitForAll | • | • | • | • |  | • |
| Sys8 | Kitchen and Cooking |  |  | • |  |  |  |
| Sys9 | Find the Pair |  |  |  |  |  |  |
| Sys10 | VAP-M | • | • | • |  |  |  |
| Sys11 | whack-a-mole |  |  | • |  |  |  |
| Sys12 |  |  |  |  |  |  |  |
| Sys13 | Smartkuber | • | • | • | • | • | • |
| Sys14 |  | • | • |  | • | • |  |
| Sys15 |  |  |  |  |  |  |  |
| Sys16 |  |  |  |  |  |  |  |
| Sys17 | Dr. Solitaire | • | • | • | • |  |  |
| Sys18 |  | • | • | • | • | • | • |
| Sys19 | Counting Sheep | • |  |  | • |  |  |
| Sys20 | Panoramix | • | • | • | • |  |  |
| Sys21 | Neuro-World | • |  |  | • |  |  |
| Sys22 |  |  |  |  |  |  |  |
| Sys23 | Virtual ADL+House | • | • | • | • | • |  |
| Sys24 |  | • | • | • | • |  |  |
| Sys25 | RE@CH | • |  | • |  |  |  |
| Sys26 | WarCAT |  |  |  |  |  |  |
| Sys27 |  |  |  |  |  |  |  |
| Sys28 | Holey Moley |  |  |  |  |  |  |
| Sys29 | Hit-the-ball |  |  |  |  |  |  |
| Sys30 | Virtual Supermarket | • | • | • | • |  |  |
| Sys31 |  | • | • |  | • |  |  |
| Sys32 | VSIDCS | • | • | • | • | • | • |
| Sys33 | Lucy | • |  |  | • |  |  |
| Sys34 | Quick, Draw! |  |  |  |  |  |  |
| Sys35 |  | • | • |  |  |  |  |
| Sys36 | COGNIPLAT | • | • | • | • | • | • |
| Sys37 | Pac-man |  |  |  |  |  |  |
| Sys38 | CogWorldTravel | • | • | • | • | • |  |
| Sys39 |  | • | • | • | • |  |  |
| Sys40 | Neurocity | • |  |  |  |  |  |
| Sys41 | Minecraft |  |  |  |  |  |  |
| Sys42 | Seas the Day |  |  |  |  |  |  |
| Sys43 |  |  |  |  |  |  |  |
| Sys44 |  | • | • | • | • |  |  |
| Sys45 |  |  |  |  |  |  |  |
| Sys46 | BrightArm | • |  | • | • |  |  |
| Sys47 | RehabCity |  | • | • | • |  |  |
| Sys48 | The Ryokansan | • |  | • | • |  |  |
| Sys49 | X-Torp |  |  |  | • |  |  |
